# The Barrow Innovation Center: A Novel Program in Neurosurgery Resident Education and Medical Device Innovation

**DOI:** 10.7759/cureus.2142

**Published:** 2018-02-02

**Authors:** Michael A Bohl, Michael A Mooney, John Sheehy, Clinton D Morgan, Michael J Donovan, Andrew Little, Peter Nakaji

**Affiliations:** 1 Department of Neurosurgery, Barrow Neurological Institute, St. Joseph's Hospital and Medical Center, Phoenix, USA; 2 Intellectual Property, Dignity Health

**Keywords:** innovation, interdisciplinary training, resident education

## Abstract

Medical innovation is the application of scientific knowledge and problem solving for the betterment of the human condition. Every great advancement in the field of neurosurgery can be traced back to a novel surgical procedure or technology that challenged existing standards of care. Considering the critical importance of innovation to the advancement of neurosurgery, and a surprising lack of formal training in innovation among residency programs, we sought to create a residency training program in neurosurgical innovation. Neurosurgery residents at the authors’ institution envisioned the creation of a program that contained all the necessary equipment, personnel, and information required to bring their ideas from theoretical concepts to functional devices implemented in a clinical setting. The Barrow Innovation Center was established as a result. The center currently comprises a rapid prototyping laboratory and several collaborative partnerships between neurosurgery residents, patent law students, and biomedical engineering students. The creation of this model was guided by an overarching mission to educate the next generation of neurosurgical innovators. With modest start-up capital and strong faculty and institutional support, the center has grown from a simple idea to a multistate, multidisciplinary collaboration in just 18 months; it has generated substantial intellectual property, educational opportunities, and a new business entity. We hope that by continuing to advance the Barrow Innovation Center and its core mission of innovation education, we will advance the field of neurosurgery by providing the next generation of surgeon-scientists with the skills, knowledge, and opportunity needed to revolutionize the field.

## Introduction

From the first craniotomies performed with stone tools during the Neolithic Era to Cushing and Bovie’s development of electrocautery to the introduction of the operating microscope and microsurgical instruments, the field of neurosurgery has been a catalyst for collaborative advances in surgical and engineering technologies [1-4]. Such collaboration has, in turn, advanced the neurosurgeon’s ability to treat patients with diseases once thought to be untreatable. The common thread to neurosurgical advances is a willingness to innovate. Innovation is defined as “the act or process of introducing new ideas, devices, or methods” [5]. As Krummel [6] explained, “A scientist seeks understanding, an inventor seeks a solution, an innovator seeks an application.” Innovation is therefore inventive science in action; it is the application of scientific knowledge and problem solving to the betterment of the human condition. In other words, innovation is at the core of what it means to be a physician, and especially a neurosurgeon.

Every great advancement in the field of neurosurgery can be traced back to a novel surgical procedure or technology that challenged existing standards of care. This is, in part, what makes innovation so difficult; it “runs against [the] gradient of [the status quo] and frequently seems self-destructive,” as acceptance of the innovation requires rejection of the old standard [6]. Modern surgeon-innovators face the additional challenge of exponential growth of technology in the modern era; this rapid growth can limit access to new technologies to engineers and other non-medical professionals. The net result of these challenges is the progressive sidelining of physicians from the process of medical innovation. In their 2016 best-practice recommendations on this issue, Silver et al. [7] summarized this growing problem: “Physicians and other healthcare professionals are often the end users of medical innovation; however, they are rarely involved in the beginning design stages. Too often this results in ineffective and inefficient solutions with poor adoption rates.” Others have recognized the enormous potential of modern technologies to improve the quality and cost of healthcare, but note that innovation has become largely sequestered in medical device companies where ideas originate from engineers with limited knowledge of the clinical complexities of any given field. This model of innovation typically results in incremental advances on existing products that may generate heavy capital investment but also increased treatment costs and limited population impact. The result has been an inefficient, costly, and slow development process for bringing new innovative medical technologies to the patients who need them [7-10].

To break this cycle, young neurosurgeons must actively involve themselves in the process of medical innovation. In his 2006 comments on the coming challenges in resident education posed by modern trends in medical innovation, Satava [11] wrote, “It is mandatory to instill in our students a feeling of urgency, a sense of magnitude, and an acceptance of obligation to not only our patients and the profession, but [also to] all of humankind…. To ignore this burden is to abdicate to those (such as politicians and lawyers) who blur the sacrifice of idealism with their own reality of self-interest.” More than 10 years later, what can we as neurosurgeons say about our commitment or our sense of urgency to train the next generation of innovators?

In the last decade, the US Accreditation Council for Graduate Medical Education and the Neurosurgery Residency Review Committee have implemented a number of important changes to neurosurgery residency training, including the creation of training milestones and the formalization of training requirements. However, none of these milestones or training requirements make any mention of innovation skills acquisition [12, 13], nor do the standard neurosurgical curricula in the United Kingdom or Canada [14, 15]. Some universities have taken up the task by offering one- or two-year master’s degree programs in medical device innovation, during which multidisciplinary teams work intensively for the duration of their degree program on identifying a problem, brainstorming solutions, and then building and testing prototypes [16-19]. Other universities, such as the Massachusetts Institute of Technology, have taken an even more intensive approach by hosting “healthcare hackathons,” typically two-day events where clinicians and engineers come together to work on identifying problems and brainstorming solutions [7, 20]. These master’s degree programs and hackathons have successfully generated important new technologies, and most importantly have proven the utility of bringing clinicians into the innovation process as early as possible. However, they require the resident surgeon to leave clinical training to receive an education in device innovation, they exist on an artificial timeline of need (i.e., problems are actively sought for the sake of course completion, rather than identified in the process of clinical practice), and they represent an artificial period during which the surgeon has access to all the resources and collaborators necessary to innovate successfully.

Considering the critical importance of innovation to the advancement of neurosurgery, and a surprising lack of formal training in innovation among residency programs, we sought to create a residency training program in neurosurgical innovation. This program is a resident-initiated, faculty-supported effort to formalize training in modern healthcare innovation. Innovation in this sense is broadly defined to include the development of novel surgical procedures, devices, medical treatments, and diagnostic modalities. The following report describes the development, structure, and goals of this integrated residency training program in surgical innovation, now known as the Barrow Innovation Center.

## Materials and methods

Development of the Barrow Innovation Center

Neurosurgery residents at the authors’ institution were inspired to develop a framework for the pursuit of innovative ideas and envisioned the creation of a virtual center that contained all the necessary equipment, personnel, and information required to bring their ideas from the “napkin drawing” phase to the bedside. The foundational elements included seed funding for a rapid prototyping laboratory, legal collaboration, engineering collaboration, and an intellectual property-sharing framework.

Seed funding

A grateful family who had received care at the authors’ institution donated money toward funding resident-initiated research [21]. The residents submitted a grant request and were awarded $5,000 for the creation of the innovation center from the Lisa Family Foundation. Shortly after this donation was made, the Barrow Neurological Foundation made a matching donation. This money was used toward the creation of a rapid prototyping center, equipped with three-dimensional (3D) modeling, 3D scanning, and 3D printing hardware.

Legal collaboration

More important than the seed funding, however, was the help given by the Lisa Family Foundation to initiate a collaboration between the neurosurgery residents and the Arizona State University (ASU) patent law students. As part of their coursework, the ASU law students run a patent law clinic in which they provide legal advice and assistance to clients with patentable ideas. Neurosurgery residents effectively began acting as clients of this student-run patent law clinic, presenting their ideas to the law students and their professors, receiving feedback, and then working together to draft provisional patent applications. The law students receive a grade and course credit for their work with the residents, and the residents not only are assisted in the drafting of a provisional patent application but also learn the basics of patent law through their work with the law students. This collaboration between the neurosurgery residents and law students proved to be quite productive, resulting in institutional support from both the hosting hospital and ASU and generating favorable publicity on this unique partnership [21].

Engineering collaboration

The success of the neurosurgery resident collaboration with the ASU Patent Law Clinic led to the creation of additional collaborative partnerships. The neurosurgery residents, who were frustrated with their inability to efficiently prototype and test their novel designs, believed that a partnership with engineers was the next foundational element for the center. The resident and faculty leadership of the center contacted local and out-of-state academic partners, and after several months had initiated collaborations with several biomedical engineering programs, including those at ASU, California Polytechnic State University, and Texas A&M. Similar to the residents’ partnership with the ASU law students, these partnerships entailed engineering students working directly with neurosurgery residents to exchange thoughts on specific clinical problems and potential solutions. Residents’ ideas for novel medical devices were provisionally patented and then presented to biomedical engineering programs. These programs then offered their students the opportunity to work on designing, prototyping, and testing these devices for credit toward their degree. In turn, residents had an opportunity to work directly with engineers on the design and testing of their devices, and to learn about the engineering process.

Intellectual property agreements

The residents’ home institution was willing to engage other institutions and to sign contracts that formalized collaborative relationships in the development of intellectual property. This engagement was a critically necessary step as it required each institution to agree on how to handle the intellectual property rights generated through these collaborations. Consistent with most residency programs, the authors’ institution owns all intellectual property generated by residents during their training and incentivizes innovation through a 40-40-20 sharing agreement (40% to the resident inventor, 40% spent within the institution per the resident’s discretion, and 20% to the institution). As such, it was critically important for the institution to agree to negotiate with the center’s collaborative partners on the intellectual property rights of devices developed through this program. With these agreements in place, the center then had all the necessary infrastructure and collaborators for idea development: neurosurgery residents generating ideas and rapidly prototyping them in their 3D printing laboratory, patent law students identifying and helping to protect patentable ideas, and engineers designing and formally prototyping the inventive solutions. Thus, the current model of the Barrow Innovation Center was formed.

Mission statement and functional description

The creation of the Barrow Innovation Center model was guided by an overarching mission to educate the next generation of neurosurgical innovators. This educational mission is founded on the beliefs that innovation education should be included in the day-to-day education of residents, the barrier to entry as an innovator should be as low as possible, and maintaining a spirit of collaboration and idea sharing to advance the realization of an inventive idea should be an encouraged norm. These beliefs are consistent with the primary goal to educate neurosurgeons in the process of innovation in as risk-free an environment as possible, while also providing unique practical education opportunities to our collaborators in law and engineering. Achieving this primary mission will have the secondary effect of advancing health sciences technology, and subsequently patient care, through the invention, prototyping, and marketing of novel medical devices.

**Figure 1:**
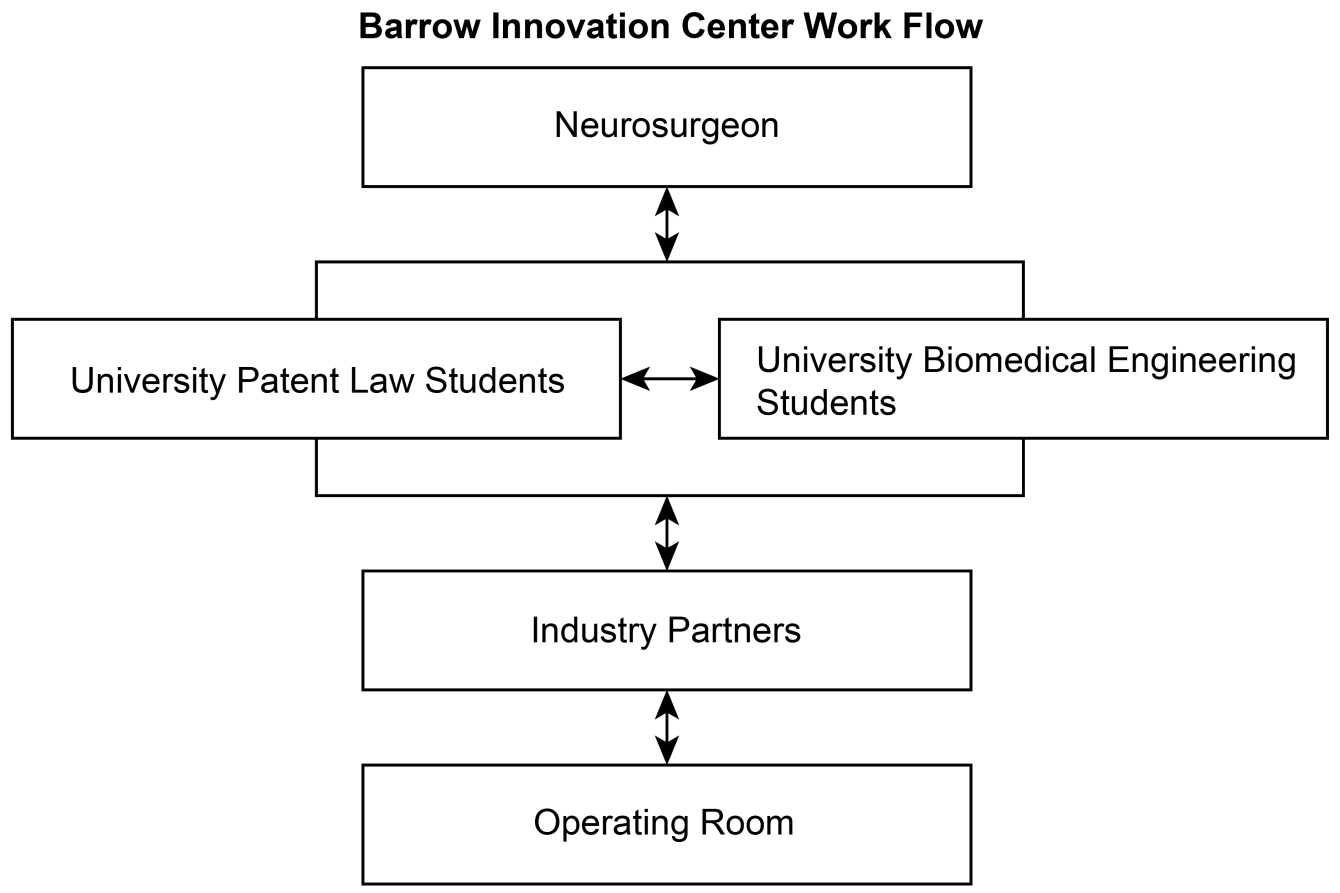
Barrow Innovation Center Work Flow. This diagram depicts the work flow of ideas through the center. Ideas are generated by, and introduced to, the center by neurosurgeons (primarily residents). At monthly meetings, these ideas are discussed by fellow surgeons, patent law students, and engineers. Incomplete ideas are sent back to the presenting neurosurgeon for more development, whereas good ideas are distributed to patent law students and engineering students. Products that have had provisional patent applications filed by the law students and functional prototypes developed by the engineering students are then presented to industry partners. Used with permission from Barrow Neurological Institute, Phoenix, Arizona.

We strive to achieve these goals by integrating innovative thinking and education throughout the standard neurosurgical curriculum. During surgical procedures and hospital rounds, residents are encouraged to think creatively about new solutions to the problems they frequently encounter. These ideas are then presented at monthly meetings, attended by neurosurgical faculty and residents, patent law students and faculty, and engineering students. During these meetings, ideas are briefly presented and discussed openly among the attendees (i.e., in a legally safe environment via established non-disclosure agreements). Neurosurgeons provide clinical critiques and recommendations, patent law students discuss potential problems with the design or known prior art in the field, and engineers discuss potential designs or alternative solutions. Promising ideas are assigned immediately to a law student and engineer to begin work, whereas ideas that require further development are recommended for presentation at a future meeting. After new ideas are discussed at the monthly meetings, existing projects are briefly addressed to ensure that steady progress is being made and to address any identified roadblocks. Furthermore, a brief didactic session is held each month, led by a patent law student addressing an important aspect of basic patent law. Ad hoc meetings between neurosurgery residents, patent law students, and engineers also occur between monthly meetings, as needed, to maintain progress. Figure 1 depicts the flow of ideas and work through the innovation center.

These educational opportunities have further opened up positions of mentorship and leadership for neurosurgery residents within partner universities. Many of the engineering students formally list their neurosurgeon partners as project mentors for their coursework or honors thesis statements, thereby opening the door for resident participation in positions of academic leadership.

In addition to these meetings, the center also hosts expert workshops on topics related to medical innovation. These are typically taught by experts in the field and, thus far, have included the chief executive officer of a mid-size neurosurgical instrument company discussing the process of approaching industry with novel ideas and an internationally recognized patent lawyer teaching “How to write a provisional patent in three hours or less.”

## Results

Current status

The Barrow Innovation Center opened its rapid prototyping laboratory and began its first semester of work with the ASU Patent Law Clinic in January 2016. In our first 18 months, we have signed formal agreements of collaboration with three university systems, two of which are out of state. We have provided practical training opportunities to more than 10 patent law students and more than 50 biomedical engineering students, and we have placed neurosurgery residents as formal university mentors for 10 separate senior engineering program theses. Neurosurgery residents working through the center have filed 12 provisional patents and achieved sufficient institutional funding and support to hire outside counsel for drafting and submitting two Patent Cooperation Treaty patent applications. The center has supported the development of 10 novel surgical procedures for bone grafting during spinal reconstructions through its partnership with the home institution’s cadaver laboratory; seven of these procedures have been performed in 14 consenting patients. Finally, the center has helped launch the formation of a company in which a neurosurgery resident is a cofounder [22]. The idea for this company and the novel postoperative care products it sells were presented at one of the monthly innovation center meetings, where the company received legal counsel on trademarking and brand protection through the ASU Patent Law Clinic and acquired its first customer through the resident’s home institution. This company has since grown to include 10 customers in two states and is planning a clinical trial to evaluate the effectiveness of its product in the coming months.

## Discussion

Considering the modest start-up capital required to begin this program, we believe that these accomplishments serve as further proof of concept for the utility of integrating innovation education programs into neurosurgical training. Because of the results achieved thus far, the authors’ home institution, the family that originally invested in the formation of the center, and the neurosurgical faculty have pledged their continued support of this effort through the creation of two formal fellowships to support the center. One fellowship is focused on engineering and the other on patent law. These fellowships will be formal, one-year programs during which the selected engineering and law school graduates will work intensively with the neurosurgery residents through the center. The engineering fellow will run the rapid prototyping and 3D printing laboratories (the clinical and research utility of which has well outgrown its original intent), as well as oversee the recent expansion of the innovation center’s 3D printing laboratory into its own formal research program. The legal fellow will act as a direct liaison with the residents, engineering students, and hospital, working to write nonprovisional quality patent applications with the neurosurgery residents, and helping to identify ideas for which it is worth pursuing Patent Cooperation Treaty patent protection. Table 1 summarizes the first- and second-year goals stated during the center’s first month of operation, as well as the achievements of our first 18 months of operation and our goals for the coming year.

**Table 1:** Barrow Innovation Center’s one- and two-year goals and 18-month achievements.

One- and two-year goals established during the first month of operation	Goal achieved? (yes/no)	Additional accomplishments achieved within first 18 months of operation	New goals for the coming year
Establish partnership with ASU Patent Law Clinic	Yes	Partnerships established with two biomedical engineering schools: California Polytechnic State University and Texas A&M 12 provisional patent applications filed Four additional patent applications currently being prepared Patent Cooperation Treaty applications prepared and submitted for two projects Eight neurosurgery residents collaborated with and, in some cases, mentored >10 law students and ~50 biomedical engineering students 10 novel surgical procedures developed 13 patients treated using these novel surgical procedures A company was formed, trademarks were filed, and an industry partner was identified for the company’s launch The Lisa Family Foundation donated funding to support the creation of two fellowships in the center (engineering and patent law) 3D printing laboratory prototyped >10 functional prototypes 3D printed prototypes have routinely been tested in the cadaver and spine biomechanics laboratory 3D printing laboratory grew to include two additional printers and 0.8 FTE staff engineer Industry partnerships with medical device companies are actively being explored Fact-finding trip taken to the Jacob’s Institute in Buffalo, NY	Introduce the first prototyped device to the operating room Establish field-specific industry partners for development of products Have the first device manufactured by an industry partner Host the first annual Barrow Innovation Center conference Receive the first non-provisional patent for a device developed at the center
Establish partnership with ASU Biomedical Engineering	Yes		
Submit three provisional patent applications in the first year	Yes		
Submit six provisional patent applications in the second year	Yes		
Establish a functional 3D printing and prototyping laboratory	Yes		
3D print a functional prototype in the first year	Yes		
Test a functional prototype in the cadaver lab in the first year	Yes		
Establish headquarters on hospital campus	No		
Establish industry partners for development of products	No		

We believe that the incorporation of innovation training throughout the neurosurgical residency training curriculum is novel among surgical innovation programs. For example, numerous universities offer surgical innovation programs for general surgery residents, medical students, engineers, and faculty. These programs require the surgery residents to set aside three months to two years of elective time during which they work intensely on a single project [23-27]. Although these programs are effective at educating a broad variety of students on the process of medical innovation, they perhaps miss an opportunity to properly prepare surgical residents for innovating once they enter clinical practice. Clinical practice does not afford long periods of time devoted to intensive work on a single clinical problem. It is even less probable that a large, multidisciplinary team of clinicians, engineers, and attorneys would be able to work intensely on a shared project for an extended period. Therefore, it is critical to the training of future innovators to include an education on how to incorporate innovation into daily practice and how to find engineering, legal, and industry support once outside the university setting. The Barrow Innovation Center intends to incorporate innovation training throughout the seven-year resident curriculum and to educate residents on how to evaluate ideas and seek appropriate legal and engineering support.

Future goals

After 18 months of operation, we are looking forward to and planning for the continued growth of the innovation center. Current priorities include the formation of industry collaborations, such that select industry partners will have an opportunity to collaborate with residents early on in the innovation process. We are also planning on hosting the first of what is intended to be an annual innovation conference, to which we will invite national leaders in medical innovation and where we will discuss emerging new technologies, explore areas of great need and great promise, and conduct laboratory demonstrations of prototyped and provisionally patented projects from the center. We are also planning to expand and move the center into its own space on the hospital campus. The founders of the center recently visited a pillar of innovation center excellence, the Jacobs Institute at the Gates Vascular Institute in Buffalo, New York. We hope to model our innovation center along the lines of the Jacobs Institute in that we envision a workspace equidistant from clinical and research areas to foster rapid translation of solutions to clinical problems through the center and into the laboratories for prototyping and testing, with subsequent translation back to the operating room or bedside shortly thereafter. Although this goal is certainly a long-term goal, the center is being included in future plans for building expansion and renovation.

## Conclusions

Nearly 100 years ago, William Halsted said, “The art of surgery is not yet perfect and advancements now unimaginable are still to come. May we have the wisdom to live with this with grace and humility.” The process of innovation is difficult in large part because it requires the grace and humility to acknowledge the shortcomings of our profession, and the bravery to bring new ideas, devices, and methods to our patients. To best prepare the next generation of neurosurgical innovators, we believe it is necessary to integrate this training throughout residency, and to furthermore reduce all possible barriers to resident entry into the innovation field. The formation and early record of productivity for the Barrow Innovation Center mark it as the first program of its kind to become integrated into a standard neurosurgical curriculum. It is our hope that by continuing to advance the center and its core mission of innovation education, we will advance the field of neurosurgery by providing the next generation of surgeon-scientists with the skills, knowledge, and opportunity needed to revolutionize the field.
